# Supplementary material for: Test of quantum thermalization in the two-dimensional transverse-field Ising model
Source: Sci Rep. 2016 Dec 1;6:38185. doi: 10.1038/srep38185 (PMC5131304; doi:10.1038/srep38185)
Supplement: Supplementary Material [file srep38185-s1.pdf]

# - Supplementary material - Test of quantum thermalization in the two-dimensional transverse-field Ising model

Benjamin Blaß[1,*] and Heiko Rieger[1,†]

[1]Theoretical Physics, Saarland University, 66123 Saarbrücken, Germany
[*]bebla@lusi.uni-sb.de
[†]h.rieger@mx.uni-saarland.de

ABSTRACT

In this supplementary material we describe the determination of the coefficients in the equations of motion of the variational parameters after the interaction and the field quenches. In case of the interaction quenches the coefficients are time-dependent expectation values which are evaluated at each time step with a single spin flip quantum Monte Carlo algorithm described in the first section. In contrast to this our ansatz for the field quenches in the ferromagnetic phase leads to a set of coupled linear differential equations of first order with constant (time-independent) coefficients which are independent of the actual quench parameters. Their determination is thus a pure combinatorics problem solved with rare event sampling described in the second section.

Coefficients of the equations of motion for the interaction quenches

The coefficients in the equations of motion of the variational parameters after the interaction quenches are time-dependent expectation values given by

$$\langle \hat{\mathscr{O}} \rangle_t = \frac{\sum_{\mathbf{x}} |\Psi(\mathbf{x},t)|^2 \mathscr{O}(\mathbf{x})}{\sum_{\mathbf{x}} |\Psi(\mathbf{x},t)|^2} \,. \tag{1}$$

We approximate them by mean values using a single spin flip Monte Carlo algorithm[1]. The statistical error of the mean values is inverse proportional to the square root of the number of samples, so that for a sufficiently large number of samples the mean values are good approximations for the expectation values.
The algorithm starts in a random configuration $\mathbf{x}_0$ of the system, i.e. the orientation of each spin (*up* or *down*) is randomly chosen. In each Monte Carlo step the flip of one single spin of the system is proposed. The acceptance probability $A(\mathbf{x} \to \mathbf{x}')$ for the transition from configuration $\mathbf{x}$ to $\mathbf{x}'$ reads

$$A(\mathbf{x} \to \mathbf{x}',t) = \min\left[1, Q(\mathbf{x} \to \mathbf{x}',t)\right] \quad \text{with} \quad Q(\mathbf{x} \to \mathbf{x}',t) = \frac{P(\mathbf{x}',t)T(\mathbf{x}' \to \mathbf{x})}{P(\mathbf{x},t)T(\mathbf{x} \to \mathbf{x}')} \,. \tag{2}$$

$P(\mathbf{x},t)$ is the probability distribution of the states of the $\mathbf{x}$-basis, which is defined by the variational state at the time t according to

$$P(\mathbf{x},t) = \frac{|\langle \mathbf{x}|\Psi(t)\rangle|^2}{\sum_{\mathbf{x}'} |\langle \mathbf{x}'|\Psi(t)\rangle|^2} = \frac{|\Psi(\mathbf{x},t)|^2}{\sum_{\mathbf{x}'} |\Psi(\mathbf{x}',t)|^2} \,. \tag{3}$$

As the operators $\hat{C}^{xx}_{\mathbf{r}}$ are diagonal in the $\mathbf{x}$-basis, the scalar product $\Psi(\mathbf{x},t)$ can be easily computed:

$$\Psi(\mathbf{x},t) = \frac{1}{\sqrt{2^N}} \exp\left(\sum_{\mathbf{r}} \alpha_{\mathbf{r}}(t) C^{xx}_{\mathbf{r}}(\mathbf{x})\right) \quad \text{with} \quad C^{xx}_{\mathbf{r}}(\mathbf{x}) = \langle \mathbf{x}|\hat{C}^{xx}_{\mathbf{r}}|\mathbf{x}\rangle \,. \tag{4}$$

The summation runs over all independent directions $\mathbf{r}$ in the lattice. Two directions are called independent, if they cannot be transformed into each other by exchanging components or altering their signs. There are up to 8 dependent directions belonging to $\mathbf{r}$. If $\mathbf{r} = (r_x, r_y)$ these directions are (r_x, r_y), $(r_x, -r_y)$, $(-r_x, r_y)$, $(-r_x, -r_y)$, (r_y, r_x), (r_y, r_x), $(r_y, -r_x)$, $(-r_y, r_x)$ and $(-r_y, -r_x)$. Their number reduces if two or more of the aforementioned vectors are identical. The number of all spin pairs

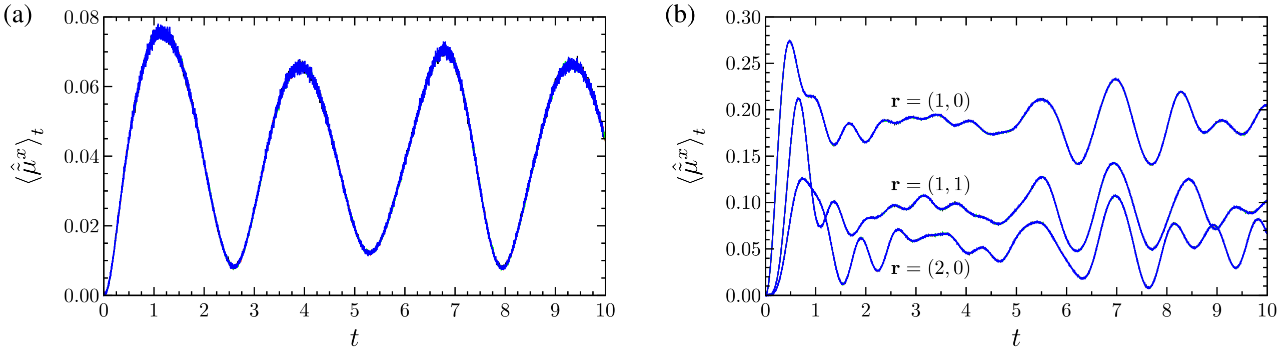

Figure 1. Time evolution of (a) the rescaled modulus of the magnetization and (b) the equal time correlation function for different $\mathbf{r}$ after the interaction quench $(0;3.5) \to (1;3.5)$ in the 16×16 system. The colour code is as follows: – nmcs = 20000, sbm = $2N$, $\delta t = 0.01$ / – nmcs = 40000, sbm = $2N$, $\delta t = 0.01$ / – nmcs = 20000, sbm = $4N$, $\delta t = 0.01$ / – nmcs = 20000, sbm = $2N$, $\delta t = 0.001$. nmcs defines the number of samples for the Monte Carlo average, sbm the number of Monte Carlo steps between two measurements and δt the stepwidth for the numerical integration with the Runge-Kutta scheme. We observe a perfect agreement between the curves.

of the systems whose distance belongs to the independent direction $\mathbf{r}$ is denoted with $N_\mathbf{r}$.
$T(\mathbf{x} \to \mathbf{x}')$ is the sampling distribution function. For the 2D-TFIM it is time-independent and inverse proportional to the number $K(\mathbf{x})$ of basis states which can be reached from the current configuration via the off-diagonal element of the Hamiltonian. $K(\mathbf{x})$ is thus for each configuration $\mathbf{x}$ equal to the number N of sites in the system.
Inserting the results for $P(\mathbf{x},t)$ and $T(\mathbf{x} \to \mathbf{x}')$ into equation (2), we find

$$A(\mathbf{x} \to \mathbf{x}',t) = \min\left[1, \exp\left\{2\sum_{\mathbf{r}} \alpha_\mathbf{r}^R(t)\left(C_\mathbf{r}^{xx}(\mathbf{x}') - C_\mathbf{r}^{xx}(\mathbf{x})\right)\right\}\right] \tag{5}$$

with $\alpha_\mathbf{r}^R(t)$ the real part of $\alpha_\mathbf{r}(t)$. The acceptance probability $A(\mathbf{x} \to \mathbf{x}')$ of a proposed Monte Carlo step thus does not depend explicitly on the Hamiltonian of the system, but the parameters of the Hamiltonian just enter via the time-evolved state defining the probability distribution.
The initial configuration $\mathbf{x}_0$ of the system, i.e. the orientation of each individual spin, is randomly chosen. For this configuration the relative orientations of all possible spin pairs are determined and used to compute the correlation functions $C_\mathbf{r}^{xx}(\mathbf{x}_0)$ for each independent direction $\mathbf{r}$. In each Monte Carlo step the flip of a randomly chosen spin of the system is proposed. Let its position be $\mathbf{R}$. To compute the acceptance probability $A(\mathbf{x} \to \mathbf{x}')$ for the transition from the configuration $\mathbf{x}$ to the proposed configuration $\mathbf{x}'$, which differs from $\mathbf{x}$ only by the inverted orientation of the spin at the position $\mathbf{R}$, the correlation functions $C_\mathbf{r}^{xx}(\mathbf{x}')$ of the proposed configuration $\mathbf{x}'$ are needed. They can be efficiently computed flipping the spin at position $\mathbf{R}$ and then computing the relative orientations between the flipped spin and all other spins of the system. In this way the $C_\mathbf{r}^{xx}(\mathbf{x}')$ can be computed from the already known $C_\mathbf{r}^{xx}(\mathbf{x})$. If the step is accepted, the configuration $\mathbf{x}$ is replaced by $\mathbf{x}'$ and the correlation functions $C_\mathbf{r}^{xx}(\mathbf{x})$ by the $C_\mathbf{r}^{xx}(\mathbf{x}')$.
As the initial configuration is randomly chosen, we wait $10N$ Monte Carlo steps for the system to equilibrate before the first measurement. Measurement means that the expectation values of the operators in the current configuration of the system are used in the computation of the mean values. After the equilibration measurements are done each $2N$ Monte Carlo steps so that the generated samples are independent. We do 20000 measurements, which we split up in 20 independent Monte Carlo runs with different seeds and 1000 samples each. The approximation of the expectation value is thus the average over 20 Monte Carlo runs. The time propagation, i.e. the solution of the equations of motion after the determination of their coefficients, is done with a fourth order Runge-Kutta scheme with a stepwidth of 0.01.
To check the convergence of the Monte Carlo algorithm and its accuracy we used different seeds for the Monte Carlo runs, i.e. different initial configurations and different sequences, and compared the results. In addition we changed the number of Monte Carlo steps between two measurements to check that the samples used to compute the mean values are really independent. We then changed the number of samples for the Monte Carlo averages to reduce their error. Finally we reduced the stepwidth of the Runge-Kutta scheme. We found that for the above values of the parameters of the Monte Carlo algorithm the results are robust with respect to the described tests. This is illustrated in Figure 1 for the rescaled modulus of the magnetization and three different equal time correlation functions of the 16×16 system (the largest system size we consider) and the quench $(0;3.5) \to (1;3.5)$ (the strongest interaction quench we consider). Using the aforementioned parameters the CPU time for the 16×16 system is approximately 400 seconds for each time step on one core of an Intel Xeon CPU E5-2680 v2 running at

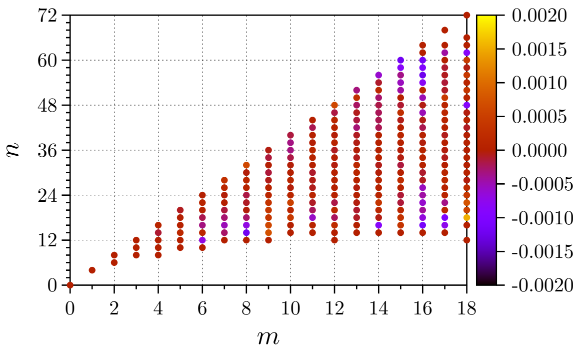

Figure 2. Relative error of the results of the RES with respect to the exact results for the $N_{m,n}$ for a 6×6 system. The values are mean values of 10 Monte Carlo runs of $2 \cdot 10^8$ Monte Carlo samples each. Values for $m > \frac{N}{2}$ are symmetric with respect to $m = \frac{N}{2}$.

2.8 GHz. This includes the single spin flip Monte Carlo algorithm and the numerical integration with the Runge-Kutta scheme. The Monte Carlo algorithm to compute the coefficients in the equations of motion of the variational parameters scales with $\mathcal{O}(N^2)$ as there are $2N$ Monte Carlo steps between two measurements and in each Monte Carlo step the relative orientation of the spin which is proposed to be flipped to all other spins of the system has to be determined.

Coefficients of the equations of motion for the field quenches

The determination of the $N_{m,n}$ and $T_{m,n;m',n'}$ to compute the $t_{m,n;m',n'}$ in the equations of motion of the variational parameters after the field quenches is a pure combinatorics problem, in which each spin configuration has the same probability given by the inverse dimension of the Hilbert space. As there are large differences between the dimensions $N_{m,n}$ of the subspaces (m,n), standard Monte Carlo methods are not able to reach low-dimensional subspaces in an efficient way and return reliable results. For this reason we apply rare event sampling (RES)[2]. Its idea is to first generate samples according to the original distribution, the majority of which will belong to subspaces of a large dimension. Then the sampling distribution is changed so that its maximum is shifted to subspaces for which only a small number of samples is generated according to the original distribution. As the results of the unbiased and the biased sampling have to agree, the estimators for the subspaces of small dimensions can be determined choosing the a priori unknown partition function under the constraint to minimize the difference between the results of the unbiased sampling and the biased sampling for regions of the Hilbert space in which both results are reliable. This procedure is repeated until the whole Hilbert space has been covered iteratively.

The $N_{m,n}$ and $T_{m,n;m',n'}$ are determined separately for each value of m. We compute them as mean values over several independent Monte Carlo sequences with different seeds and different initial states and use the ratio of the standard deviation and the mean value to decide whether the results are reliable or not. Each Monte Carlo sequence consists of one unbiased Monte Carlo run and several biased Monte Carlo runs. The number of biased Monte Carlo runs is a priori unknown, as during the Monte Carlo sequence new Monte Carlo runs with a changed sampling distributions are started until reliable results for all possible values of n with respect to m have been obtained. Each Monte Carlo run consists of a certain number of Monte Carlo steps, each of which proposes the interchange of the position of one spin up and one spin down, thus not altering m but only n. The number n_{new} of kinks in the proposed configuration is computed from the number n_{old} of kinks in the current configuration. In order to do so only the nearest neighbours of the spin down and the spin up whose positions are proposed to be interchanged have to be considered to determine how the number of kinks in the system would be changed by the proposed Monte Carlo step. For the unbiased sampling each Monte Carlo step is accepted. In the biased sampling a potential $V(n)$ and an artificial inverse temperature β are introduced. $V(n)$ depends on a parameter n_0, which shifts the position of the maximum of the distribution. β changes the width of the distribution. The acceptance probability of a step from a configuration with n_{old} kinks to a configuration with n_{new} kinks is for the biased sampling

$$p_{\mathrm{accept}} = \min\left\{1, e^{-\beta \Delta V}\right\} \quad \text{with} \quad \Delta V = V(n_{\mathrm{new}}) - V(n_{\mathrm{old}}) \,. \tag{6}$$

The potential $V(n)$ can be chosen freely, as in the last step of a Monte Carlo run the results according to the unbiased distribution are determined. Thus the focus in the choice of $V(n)$ lies on a high efficiency of the algorithm, which is mainly a question of experience. After tests with different potentials we have chosen $V(n)$ in the following way: Let $n^{\star}_{m}$ be the most probable

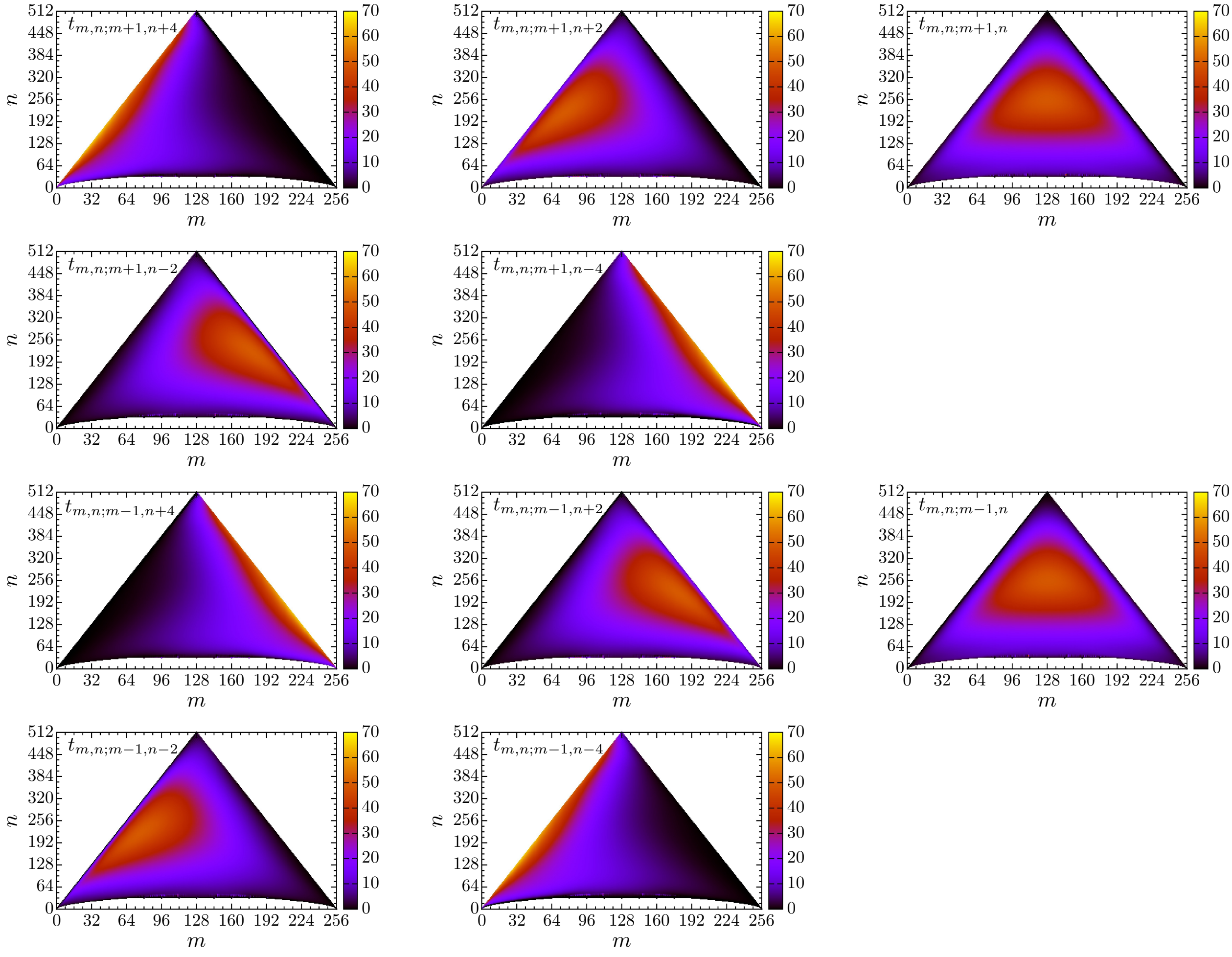

Figure 3. Coefficients $t_{m,n;m',n'}$ for the equations of motion of the variational parameters after the field quenches for the 16×16 system. One observes $t_{m,n;m+1,n+\Delta n} = t_{m,n;m-1,n-\Delta n}$.

number of kinks for a given value of m according to the unbiased distribution. We then use

$$V(n) = \begin{cases} 0 & \text{if } n \leq n_0 \\ (n-n_0)^2 & \text{else} \end{cases} \tag{7}$$

to sample $N_{m,n}$ and $T_{m,n;m',n'}$ for $n < n_m^\star$ and

$$V(n) = (n-n_0)^2 \tag{8}$$

for $n > n_m^\star$.

β and n_0 are changed after each Monte Carlo run to shift the distribution further into regions of the Hilbert space for which results are still missing in the following way: If in the last Monte Carlo run no new reliable results have been obtained, β is increased to narrow the distribution. Otherwise β is kept constant and n_0 is set just below or above the smallest or largest n respectively for which the results have been accepted. This procedure is repeated until for the considered value of m for all allowed values of n the results for $N_{m,n}$ and $T_{m,n;m',n'}$ have been accepted.

We checked our results for the $N_{m,n}$ and $T_{m,n;m',n'}$ in different ways. First we used the algorithm to compute the coefficients for the system sizes 4×4 and 6×6, for which $N_{m,n}$ and $T_{m,n;m',n'}$ can also be determined exactly analyzing each state of the Hilbert space. In Figure 2 we show exemplarily the relative error of the $N_{m,n}$ determined for the 6×6 system. We observe a very good agreement with deviations only slightly larger than one per mill. The results for the $T_{m,n;m',n'}$ are of the same accuracy. For larger system sizes we checked the validity of our results increasing the number of Monte Carlo runs per sequence until the

mean values of the $N_{m,n}$ and $T_{m,n;m',n'}$ over all runs became robust against adding further values generated with different seeds. We found that 20 Monte Carlo runs with different seeds are a good choice to produce reliable results. Besides for the smallest possible value of n for a given value of m we have been able to derive analytic expressions for $N_{m,n}$ and $T_{m,n;m',n'}$. As these coefficients are the hardest to be determined with RES, a good agreement here is a strong indicator for a high accuracy of the remaining coefficients. Finally we checked that $\sum_n N_{m,n} = \binom{N}{m}$ and $T_{m,n;m',n'} = T_{m',n';m,n}$.

The results presented in the main text have been generated using the following parameters: Each Monte Carlo sequence consists of 20 runs with different seeds, that is the values of $N_{m,n}$ and $T_{m,n;m',n'}$ are mean values over 20 samples. Each Monte Carlo run consists of $2 \cdot 10^8$ Monte Carlo steps. We consider the values of $N_{m,n}$ and $T_{m,n;m',n'}$ as reliable, if the ratio of the standard deviation and the expectation value of $N_{m,n}$ is smaller than 1 per mill.

In the equations of motion of the variational parameters after the field quenches only the $t_{m,n;m',n'}$ appear. Our results for them for the 16×16 system are shown in Figure 3. The described algorithm determines all the $N_{m,n}$ and $T_{m,n;m',n'}$ to a given value of m and uses them to compute $t_{m,n;m',n'}$. The CPU time for this depends crucially on m as both the number of different values of n as well as the number of possible configurations increase with m. Due to the adaptive implementation of the algorithm the necessary number of Monte Carlo runs to produce reliable results for all the subspaces $\mathscr{H}_{m,n}$ of the Hilbert space is a priori unknown. For this reason it is hard to make an exact prediction of the total CPU time. On our machines using one single CPU core the determination of all the $t_{m,n;m',n'}$ for the value of m with the longest run time took approximately three weeks for the 16×16 system. The time per Monte Carlo run in the biased sampling is around 90 seconds. Compared to these time scales the numerical integration of the equations of motion of the variational parameters is very fast. It is done with a stepwidth of $\delta t = 0.001$ and takes approximately 20 seconds per time step for the 16×16 system.

References

1. Bishop, R. F., Farnell, D. J. J. & Ristig, M. L. Ab Initio Treatments of the Ising Model in a Transverse Field. *Int. J. Mod. Phys. B* **14**, 1517 (2000).
2. Hartmann, A. K. Sampling rare events: Statistics of local sequence alignments. *Phys. Rev. E* **65**, 056102 (2002).
